# Administration of defined microbiota is protective in a murine *Salmonella* infection model

**DOI:** 10.1038/srep16094

**Published:** 2015-11-04

**Authors:** Sarah-Lynn E. Martz, Julie A. K. McDonald, Jun Sun, Yong-guo Zhang, Gregory B. Gloor, Curtis Noordhof, Shu-Mei He, Teklu K. Gerbaba, Michael Blennerhassett, David J. Hurlbut, Emma Allen-Vercoe, Erika C. Claud, Elaine O. Petrof

**Affiliations:** 1 Dept. Medicine, Gastrointestinal Diseases Research Unit, Queen’s University, Kingston, ON, K7L 2V7, Canada; 2 Division of Gastroenterology and Hepatology Department of Medicine University of Illinois at Chicago, Chicago, IL, 60612, USA; 3 Dept. Biochemistry, University of Western Ontario, London, Ontario, N6A 5C1, Canada; 4 Dept. Pathology & Molecular Medicine, Queen’s University, Kingston, ON, K7L 2V7, Canada; 5 Dept. Molecular & Cellular Biology, University of Guelph, Guelph, ON, N1G 2W1, Canada; 6 Depts. Pediatrics and Medicine, University of Chicago, Chicago, IL, 60637, USA

## Abstract

*Salmonella typhimurium* is a major cause of diarrhea and causes significant morbidity and mortality worldwide, and perturbations of the gut microbiota are known to increase susceptibility to enteric infections. The purpose of this study was to investigate whether a Microbial Ecosystem Therapeutic (MET-1) consisting of 33 bacterial strains, isolated from human stool and previously used to cure patients with recurrent *Clostridium difficile* infection, could also protect against *S. typhimurium* disease. C57BL/6 mice were pretreated with streptomycin prior to receiving MET-1 or control, then gavaged with *S. typhimurium.* Weight loss, serum cytokine levels, and *S. typhimurium* splenic translocation were measured. NF-κB nuclear staining, neutrophil accumulation, and localization of tight junction proteins (claudin-1, ZO-1) were visualized by immunofluorescence. Infected mice receiving MET-1 lost less weight, had reduced serum cytokines, reduced NF-κB nuclear staining, and decreased neutrophil infiltration in the cecum. MET-1 also preserved cecum tight junction protein expression, and reduced *S. typhimurium* translocation to the spleen. Notably, MET-1 did not decrease CFUs of *Salmonella* in the intestine. MET-1 may attenuate systemic infection by preserving tight junctions, thereby inhibiting *S*. *typhimurium* from gaining access to the systemic circulation. We conclude that MET-1 may be protective against enteric infections besides *C. difficile* infection.

The commensal bacteria of the gastrointestinal (GI) tract play an important role in maintaining normal barrier function and intestinal homeostasis^1^,^2^,^3^, and in protecting against pathogenic infections^4^. In addition to protecting the host from colonization by newly ingested microorganisms (colonization resistance), these microbes have direct effects on host inflammatory pathways and barrier function^2^,^3^,^5^. Shifts in the composition of the gut microbiota resulting in an imbalance (‘dysbiosis’) may affect normal function and contribute to diseases such as irritable bowel syndrome^6^,^7^, inflammatory bowel disease^8^,^9^, obesity^10^,^11^, and diabetes^12^,^13^,^14^.

Dysbiosis can also increase susceptibility to enteric infections^15^,^16^. *Salmonella enterica* serovar Typhimurium (*S. typhimurium*) is an important human pathogen and major cause of diarrhea, causing over 93.8 million cases of gastroenteritis and 155,000 deaths per year^17^. Prior antibiotic use has been linked to *Salmonella* infection in humans, and pre-exposure to antibiotics, particularly within two weeks prior to *Salmonella* infection, increases the risk of invasive non-typhoidal *Salmonella* disease in previously healthy children^18^,^19^. Animal studies of *S. typhimurium* infection suggest that the composition of the gut microbiota is important in protection^4^,^20^. Mice given the antibiotic streptomycin prior to *S. typhimurium* ingestion develop acute inflammation of the gastrointestinal (GI) tract with weight loss, diarrhea, and tissue inflammation localized to the cecum, as well as systemic inflammation and extra-intestinal spread of infection to other tissues (e.g. spleen) at later time points^21^,^22^. Moreover, *S. typhimurium*-infected mice colonized with a less diverse defined minimal microbiota composed of 8 bacterial species (Altered Schaedler Flora) were colonized by *S. typhimurium* at high levels (10^8^ CFU/g) and elicited pronounced enteropathogenesis by two days post-infection^4^.

Despite growing recognition that the microbiota plays an important role in protection against enteric infections, the underlying mechanisms remain unclear. We have developed a Microbial Ecosystem Therapeutic (MET-1) which has been used previously to cure recurrent *Clostridium difficile* disease in humans^23^. MET-1 is a defined, gut-derived microbial ecosystem of 33 bacteria cultured from the feces of a healthy human volunteer. As with *S*. *typhimurium, Clostridium difficile* disease is also associated with prior antibiotic exposure. The present study sought to expand our understanding of the function of MET-1 by investigating whether pretreatment with MET-1 could protect against disease in an antibiotic-associated murine model of *S. typhimurium* infection. We hypothesized that MET-1 would attenuate *S. typhimurium* infection through effects on the host barrier function, as well as on the pathogen itself.

## Results

To demonstrate the protective effect of MET-1, we used the *S. typhimurium* colitis model in which C57BL/6 mice were gavaged with 20 mg of streptomycin following a 4 hour fast then after an additional 18 hours were gavaged with MET-1, or vehicle control (VC). Four hours later, mice were gavaged with either *S. typhimurium* (“infected”) or phosphate-buffered saline (“uninfected”). Mice were euthanized 48 hours later and samples (tissues and blood) collected for analysis. (Note: in the figures, VC indicates mice that have been treated with vehicle control and streptomycin).

### MET-1 attenuates weight loss in *Salmonella typhimurium*-infected mice

**Figure 1:**
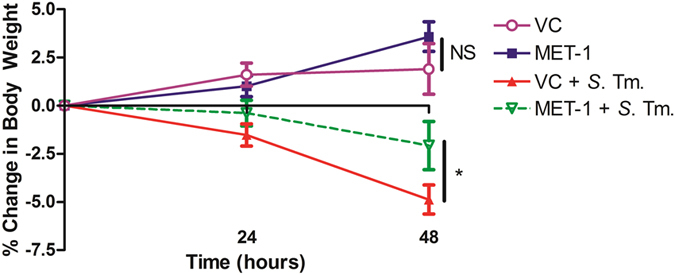
MET-1 attenuated systemic markers of disease in *S. typhimurium*-infected mice. (**a**) MET-1 attenuated weight loss in *S. typhimurium* infected mice. Following oral infection with *S. typhimurium*, mice were weighed daily and the percent change in weight from 0 to 48 hours is shown. VC = uninfected mice pretreated with vehicle control, n = 16; MET-1 = uninfected mice pretreated with MET-1, n = 16; VC + *S*. Tm. = *S. typhimurium*-infected mice pretreated with vehicle control, n = 18; MET-1 + *S*. Tm. = *S. typhimurium*-infected mice pretreated with MET-1, n = 18. Data were analyzed with a 2-way ANOVA using Bonferroni correction (*p < 0.05 at 48 hours), NS = not significant.

To determine whether MET-1 administration could confer protection against *S. typhimurium* infection, we first measured mouse body weight daily as a general indicator of the overall health of each animal^21^. *S. typhimurium*-infected mice (VC + *S*. Tm. mice) lost significant weight compared to uninfected controls (Fig. 1a). However, infected mice receiving MET-1 lost significantly less weight than mice gavaged with vehicle control (−3.50% vs. −6.83%, respectively; p < 0.05). Mice not infected with *S. typhimurium* demonstrated normal weight gain.

### MET-1 inhibits *Salmonella typhimurium* growth *in vitro* but not in the intestine

**Figure 2:**
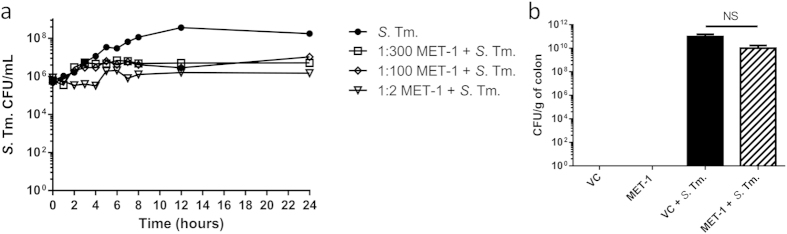
MET-1 inhibited *S. typhimurium in vitro* but did not inhibit its growth *in vivo*. (**a**) MET-1 (1:2, 1:100 and 1:300 dilutions) was incubated with *S. typhimurium* (*S*. Tm.), aliquots were withdrawn at times indicated, plated, counted, and plotted as log_10_ CFU. MET-1 inhibited the growth of *S.* Tm. relative to the *S*. Tm. control, although MET-1 was unable to completely kill *S.* Tm. even at a high (1:2) concentration. (**b**) Colons were harvested 48 hours after *S.* Tm. infection, homogenized in sterile PBS, and plated. Infected mice pretreated with MET-1 (MET-1 + *S*. Tm.) showed no significant reduction of *S.* Tm. counts in the colon (p > 0.05) compared to mice pretreated with vehicle control (VC + *S*. Tm.). *S.* Tm. was not detected in the colon of uninfected mice. Data were analyzed using a 1-way ANOVA with Bonferroni correction. VC = uninfected mice pretreated with vehicle control, n = 18; MET-1 = uninfected mice pretreated with MET-1, n = 18; VC + *S*. Tm. = *S. typhimurium*-infected mice pretreated with vehicle control, n = 22; MET-1 + *S*. Tm. = *S. typhimurium*-infected mice pretreated with MET-1, n = 21.

Studies showing that the microbiota can inhibit growth of pathogens^4^,^24^ suggest that microbe-to-microbe interactions might decrease the burden of *Salmonella* in infected animals receiving MET-1. *In vitro* time kill assays of *S. typhimurium* with MET-1 indicated that MET-1 was able to inhibit growth of *S. typhimurium*, although MET-1 was unable to completely kill *S. typhimurium* at the concentrations tested (Fig. 2a). In contrast, *in vivo* we found no evidence that MET-1 + *S*. Tm. mice had a lower colonic pathogen burden compared to VC + *S*. Tm. mice (Fig. 2b). Similar results were noted in the stool (p > 0.05, data not shown). Analysis of mouse stool by 16S rRNA gene sequencing of samples taken 48 hours post-infection comparing the data from 5 separate experiments run several weeks apart showed clustering of samples was not based on the different groups the starting microbiota in each group had little effect on the course of treatment (Figure S1). Therefore, it is unlikely that direct killing of *S. typhimurium* by MET-1 is involved in either decreased systemic inflammation or decreased pathogen translocation.

### MET-1 did not decrease intracellular invasion of intestinal epithelial cells by *Salmonella typhimurium*

**Figure 3:**
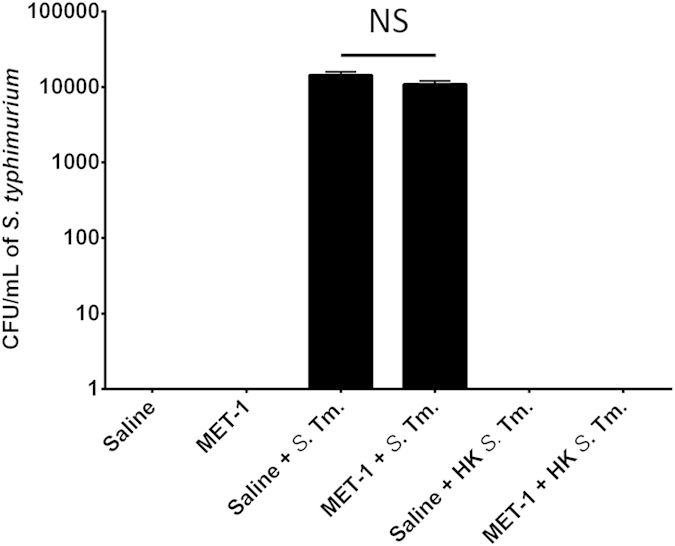
MET-1 pre-treatment does not prevent *S. typhimurium* intracellular invasion of Caco-2 epithelial cells. Live (or heat-killed) *S. typhimurium* were added to Caco-2 cell monolayers at an MOI of 100:1 and incubated for 1 hour. Extracellular bacteria were then killed by the addition of gentamicin (100 μg/mL). One hour later, Caco-2 cells were lysed and intracellular bacteria were enumerated using serial dilutions plated on MacConkey agar plates containing 100 μg/mL streptomycin. There were no significant differences between *S. typhimurium*-infected cells pretreated with MET-1 (MET-1 + Sal) or saline (Saline + Sal) (p > 0.05). No bacterial growth was seen in cell monolayers treated with saline only (Saline), MET-1 only (MET-1), heat-killed *S. typhimurium* only (Saline + HK Sal) or MET-1 pretreated cell monolayers treated with heat-killed *S. typhimurium* (MET-1 + HK Sal). Data were analyzed using a 1-way ANOVA with Bonferroni correction, n = 3, (p > 0.05) NS = not significant.

A commonly described mechanism whereby *S. typhimurium* causes infection involves active invasion of host cells^25^. This mechanism is independent of tight junction protein function and involves invasion of host cells via the Type III Secretion System (TTSS) of *S. typhimurium* to inject bacterial effector proteins, ultimately leading to internalization of the bacteria into the host cell^25^. Although major growth inhibition of *Salmonella* by MET-1 was not observed in Fig. 2, it has been reported that Clostridial species present in the fecal microbiota can inhibit the invasion virulence properties of *Salmonella typhimurium*^26^. To determine whether MET-1 could protect against inhibition of host cell invasion by *S. typhimurium*, or otherwise affect the invasive virulence capacity of *S. typhimurium*, we used an *in vitro* cell culture gentamicin-based invasion assay to determine the effect of MET-1 pretreatment on the susceptibility of Caco-2 intestinal epithelial cells to bacterial invasion by *S. typhimurium*. No differences were noted in the CFU/mL of intracellular *S. typhimurium* in either the MET-1 or saline groups treated with *S. typhimurium* (p > 0.05), indicating that bacterial invasion of intestinal epithelial cells was not prevented by MET-1 pretreatment and MET-1 did not result in statistically lower counts of *Salmonella* bacteria in this assay (Fig. 3). The controls of uninfected cell monolayer pretreated with saline, MET-1, or heat-killed *S. typhimurium* plus MET-1 did not result in any bacterial growth. These data suggest that MET-1 does not significantly affect the virulence or invasive capacity of *S. typhimurium.*

### MET-1 reduced systemic spread of infection

**Figure 4:**
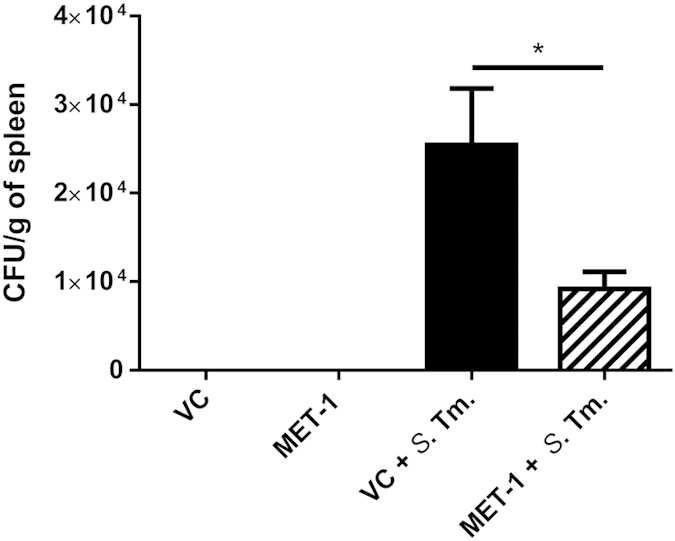
Translocation of *S. typhimurium* to the spleen was reduced in MET-1 pretreated mice. Spleens were harvested 48 hours after *S. typhimurium* infection, weighed, and homogenized in 1 mL PBS. Serial dilutions were plated on MacConkey agar plates containing 100 μg/mL streptomycin. Plates were incubated at 37 °C for 24 hrs, and the resulting colonies were counted. Infected mice pretreated with MET-1 (MET-1 + *S*. Tm., n = 13) had reduced *S. typhimurium* counts in the spleen (*p < 0.05) compared to mice pretreated with vehicle control (VC + *S*. Tm., n = 12). *S. typhimurium* was not detected in the tissues of uninfected mice pretreated with vehicle control (VC, n = 12) or MET-1 (n = 12). Data were analyzed using a 1-way ANOVA with Bonferroni correction.

Since the ability of *S. typhimurium* to translocate from the gut and cause metastatic infection is well described^21^, we sought to determine whether MET-1 would affect the ability of *S. typhimurium* to disseminate to other organs. Quantification of *S. typhimurium* in the spleen was determined by measuring the number of CFU/g of tissue, and bacterial speciation was confirmed by PCR. MET-1 + *S*. Tm mice had significantly lower *S. typhimurium* counts within the spleen compared with VC + *S*. Tm. mice (Fig. 4), indicating that MET-1 administration attenuated the ability of *S. typhimurium* to gain access to the systemic circulation. None of the uninfected mice had *S. typhimurium* present in the spleen.

### MET-1 attenuates the increase in serum cytokine levels caused by *Salmonella typhimurium* infection

**Figure 5:**
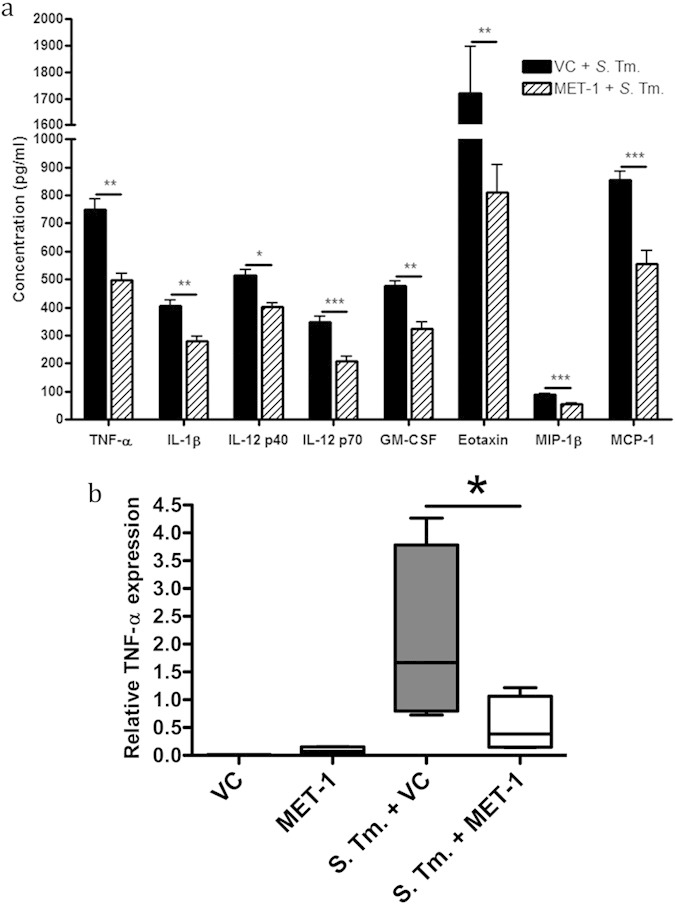
Pro-inflammatory cytokine levels were reduced in *S. typhimurium*-infected mice pretreated with MET-1. (**a**) Serum cytokine levels were measured 48 hours post-infection using a Bio-Plex Pro mouse cytokine magnetic bead kit. Of the cytokines measured, TNF-α, IL-1β, IL-12p40, IL-12p70, GM-CSF, eotaxin, MIP-1β, and MCP-1 were all significantly reduced in infected mice pretreated with MET-1 (MET-1 + *S*. Tm.) compared to infected mice pretreated with vehicle control (VC + *S*. Tm.). There were no significant differences in cytokine concentrations between uninfected mice pretreated with MET-1 or vehicle control (data not shown). Data were analyzed using a 1-way ANOVA with Bonferroni correction, n = 7 for each group (*p < 0.05, **p < 0.01, ***p < 0.001). (**b**) Cecal TNF-α expression was attenuated in *S. typhimurium*-infected mice pretreated with MET-1, as determined by quantitative RT-PCR (qRT-PCR) was performed in triplicate. The relative TNF-α expression was normalized to an endogenous control (GAPDH expression). The relative expression of TNF-α was attenuated in infected mice pretreated with MET-1 (MET-1 + *S*. Tm.) compared to infected mice pretreated with vehicle control (VC + *S*. Tm.). The level of expression in MET-1 + *S*. Tm. mice was not significantly different from uninfected mice pretreated with vehicle control (VC) or MET-1. Data were analyzed using a 1-way ANOVA with Newman-Keuls multiple comparison test, n = 4 for each group (*p < 0.05).

We next investigated whether MET-1 might attenuate the increase in systemic cytokine levels that occur after oral *S. typhimurium* infection^27^. By forty-eight hours, significant differences were seen for TNF-α, IL-1β, IL-12 p40, IL-12 p70, GM-CSF, eotaxin, MIP-1β and MCP-1 (Fig. 5a). All uninfected mice (VC mice and MET-1 mice) had similar levels of serum cytokines (p > 0.05, data not shown), while addition of MET-1 significantly reduced circulating levels of chemoattractants and inflammatory cytokines that are normally elevated in *S. typhimurium* infection. Chemoattractants for immune cells, particularly neutrophils^28^, were found at higher levels in the systemic circulation of the VC + *S*. Tm. mice compared to MET-1 + *S*. Tm. mice (Fig. 5a), suggesting that a key aspect of MET-1 action might include modulation of neutrophil mobilization.

### MET-1 attenuates local TNF-α expression induced by *Salmonella typhimurium* infection in cecum

To determine whether MET-1 also affected local pro-inflammatory cytokine expression in the intestine, we next looked at the expression of TNF-α in the cecum using quantitative real-time PCR. TNF-α is a key mediator of inflammation in the early stages of *Salmonella* infection and acts by recruiting neutrophils to the site of infection^29^,^30^. It was found that the expression of TNF-α was significantly increased in VC + *S*. Tm. mice compared to uninfected controls (Fig. 5b). However, MET-1 effectively reduced TNF-α expression in the MET-1 + *S*. Tm. treatment group. The addition of MET-1 alone had no significant effect on TNF-α expression.

### MET-1 attenuates local neutrophil infiltration in cecum induced by *Salmonella typhimurium* infection

**Figure 6:**
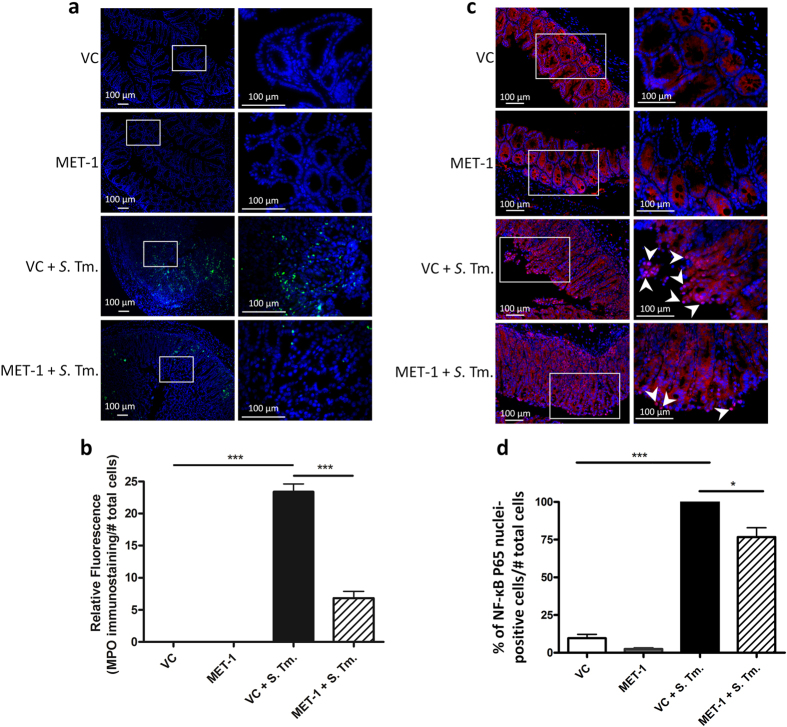
MET-1 attenuates local neutrophil infiltration induced by *S. typhimurium* infection in cecum (a) Representative images of myeloperoxidase (MPO) immunofluorescence staining for neutrophils (green) in ceca. The area in the box (left) is displayed at a higher magnification in the panels on the right. Nucleic acid was stained using Hoechst (blue). *S. typhimurium*-infected mice pretreated with vehicle control (VC + *S*. Tm.) had more abundant immunostaining in the ceca compared to uninfected controls (VC), and was significantly reduced in the ceca of infected mice pretreated with MET-1 (MET-1 + *S*. Tm. mice). No MPO staining was noted in secondary antibody control slides for each group (not shown). (**b**) Quantification of MPO immunostaining, shown by the ratio of MPO (green immunofluorescence) staining to number of cells (blue nuclei). Data analyzed using 1-way ANOVA with Bonferroni correction, ***p < 0.001. n = 4 for VC, MET-1, and MET-1 + *S*. Tm. groups, n = 3 for VC + *S*. Tm. group. 3–5 fields of view per section. (**c**) Representative images of NF-κB P65 immunofluorescence staining (red) in ceca. The area in the box (left) is displayed at a higher magnification in the panels on the right. Nuclei were stained using Hoechst (blue). VC + *S*. Tm. mice had significantly increased NFκB P65 translocation (pink immunofluorescence) into the nucleus compared with MET-1 + *S*. Tm. mice. Secondary antibody control slides for each group showed no NFκB P65 staining (images not shown). (**d**) Ratio of NF-κB P65 nuclear staining to the total number of epithelial cells per high power field. The percentage of NF-κB P65 stained cells was normalized to the VC + *S*. Tm. group. Images taken at 400× magnification from at least two sections per cecum tissue. At least five non-adjacent images were taken for each section. Images were quantified from two independent experiments and each experiment had at least three mice per group. Total number of mice for each treatment group for two independent experiments was 6- to 9. Data were analyzed using ANOVA with Bonferroni correction, (***p < 0.001 and *p < 0.05).

Localized responses of the immune system were examined in the intestinal mucosa, where infection with *S. typhimurium* causes an influx of neutrophils^31^. At 48 hours post-infection, immunocytochemistry showed increased staining for cecum MPO, a principal marker of neutrophils^32^, which was significantly reduced by treatment with MET-1 (Fig. 6a). Image analysis and quantification showed a 5-fold reduction in MPO staining in MET-1 + *S.* Tm. mice versus VC + *S*. Tm. mice (Fig. 6b). In addition, the nuclear localization of NF-κB, a key transcription factor and regulator of pro-inflammatory pathways that translocates to the nucleus when activated, was found to be reduced (Fig. 6c). The addition of MET-1 alone had no significant effect on either nuclear NF-κB or neutrophil accumulation in the ceca of uninfected mice.

### MET-1 does not enhance intestinal mucin production in cecum

**Figure 7:**
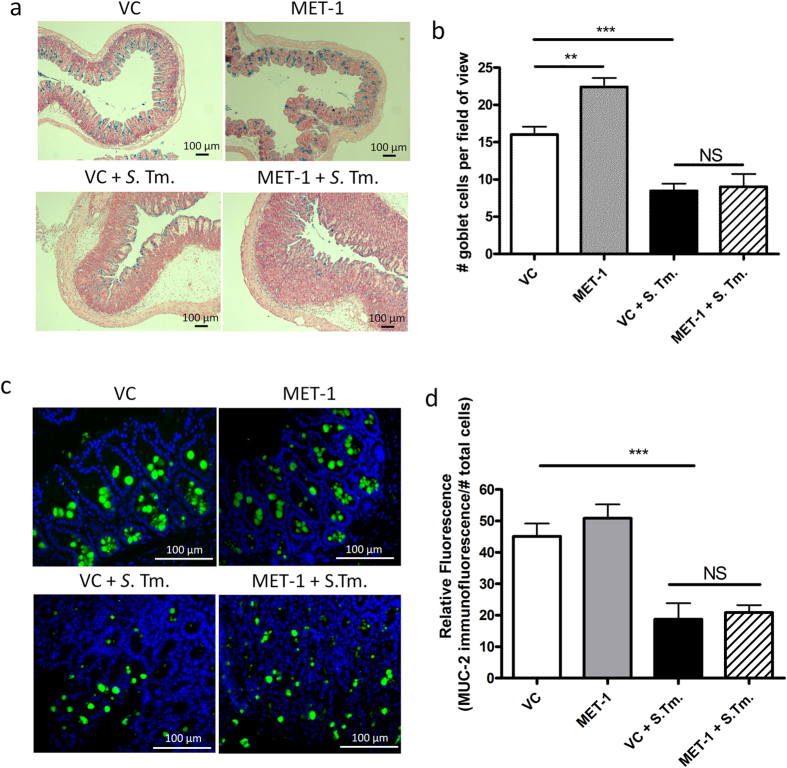
MET-1 did not enhance intestinal mucin production in ceca of *S. typhimurium*- infected mice. (**a**) Alcian blue staining (blue) of ceca fixed in 10% formalin were counterstained with nuclear red fast solution (red). (**b**) Goblet cells were enumerated from 10 random high-powered field views (400× magnification) spanning from muscularis mucosa to surface epithelium on Alcian blue stained sections. Infected mice pretreated with vehicle control (VC + *S*. Tm.) had less mucin staining than uninfected mice pretreated with vehicle control (VC + *S*. Tm.). However, there was no statistically significant difference between infected mice pretreated with MET-1 (MET-1 + *S*. Tm.) and vehicle control (VC + *S*. Tm.). (**c**) MUC2 immunofluorescence staining (green) in ceca fixed in Methanol-Carnoy’s solution. Nucleic acid was stained using DAPI (blue). Uninfected mice pretreated with VC or MET-1 had similar localization of MUC2 staining that differed from VC + *S*. Tm. mice. Again, MUC2 staining in MET-1 + *S*. Tm. mice more closely resembled VC + *S*. Tm. mice than uninfected controls. Quantification shown in (d). Data were analyzed using a 1-way ANOVA with Bonferroni correction. n = 3, (**p < 0.01, ***p < 0.001) NS = not significant.

Since MUC2 has been proposed as an important host defense mechanism against *S. typhimurium*^33^,^34^, mucin production was examined in VC + *S*. Tm. mice and MET-1 + *S*. Tm. mice. Alcian blue staining showed that MET-1 + *S*. Tm. mice did not have increased staining (Fig. 7a) or goblet cell counts (Fig. 7b) compared to VC + *S*. Tm. mice. Moreover, no differences in MUC2 immunostaining were noted in MET-1 + *S*. Tm. mice compared to VC + *S*. Tm. mice (Fig. 7c,d). These data argue against MET-1-induced differences in intestinal mucus as a likely mechanism for conferring systemic protection against *S. typhimurium*.

### MET-1 attenuates disruption of tight junction proteins in cecum caused by *Salmonella typhimurium*

**Figure 8:**
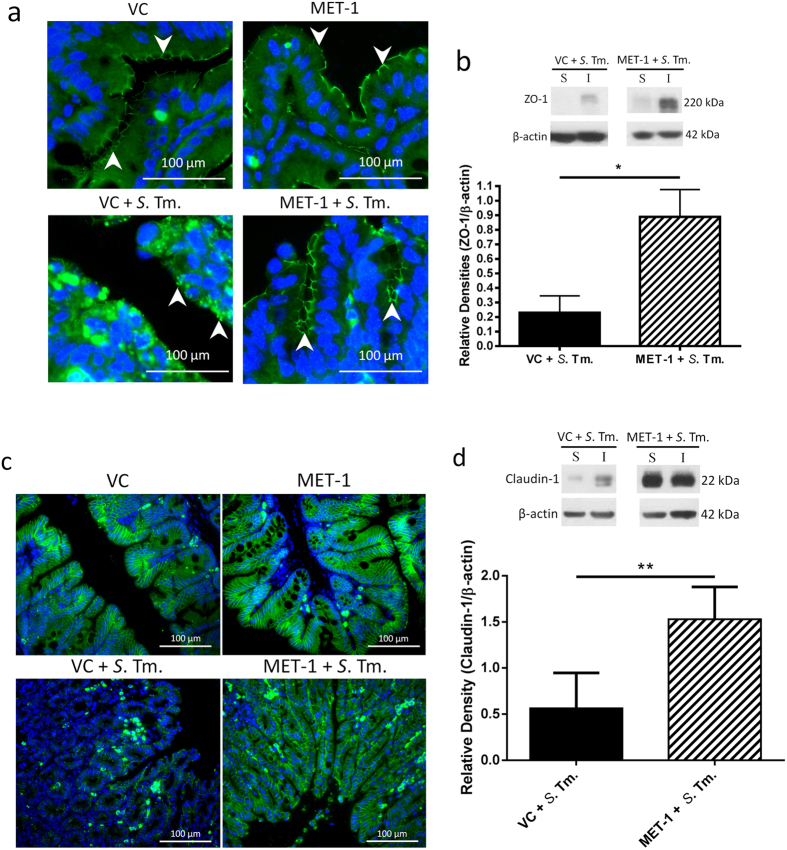
MET-1 pretreatment prevented the disruption of ZO-1 and claudin-1 cellular localization in the cecum caused by *S. typhimurium*. (**a**) ZO-1 immunofluorescence staining (green) in ceca fixed in 10% formalin (400× magnification). Nuclei were stained using Hoechst (blue). White arrows indicate green staining of ZO-1 protein at the membrane of intestinal epithelial cells. VC = uninfected mice pretreated with vehicle control; MET-1 = uninfected mice pretreated with MET-1; VC + *S*. Tm. = *S. typhimurium*-infected mice pretreated with vehicle control; MET-1 + *S*. Tm. = *S. typhimurium*-infected mice pretreated with MET-1. VC + *S*. Tm. mice had a loss of ZO-1 membrane staining that was attenuated in MET-1 + *S*. Tm. mice. (**b**) Expression of ZO-1 measured by Western blot analysis (S, Triton X-soluble fraction; I, Triton X-insoluble fraction, sample blot shown in top right of panel). Consistent with data in panel (**a**), densitometric values calculated for the ratio of ZO-1 to β-actin showed that MET-1 + *S*. Tm. mice had increased expression of ZO-1 in insoluble fractions compared to VC + *S*. Tm. mice. Data were analyzed using a 1-way ANOVA with Bonferroni correction (*p < 0.05). n = 5 mice for VC + *S*. Tm., n = 4 for MET-1 + *S*. Tm. (**c**) Claudin-1 immunofluorescence staining (green) in ceca fixed in 10% formalin (400x magnification). Nucleic acid was stained using Hoechst (blue). VC + *S*. Tm. mice displayed disaggregation and clumping of epithelial claudin-1 immunofluorescence, along with discontinuities in membrane staining and a reduction in staining intensity. This alteration in claudin-1 expression was attenuated in MET-1 + *S*. Tm. mice. (**d**) Expression of claudin-1 was measured by Western blot analysis (S, Triton X-soluble fraction; I, Triton X-insoluble fraction, sample blot shown). Densitometric values were calculated for the ratio of claudin-1 to actin and showed that MET-1 + S. Tm. mice had increased expression of claudin-1 in insoluble fractions compared to VC + *S*. Tm. mice. Data were analyzed using a 1-way ANOVA with Bonferroni correction (**p < 0.01). n = 5 mice for VC + *S*. Tm., n = 4 for MET-1 + *S*. Tm.

*S. typhimurium* causes compromise of the intestinal epithelial barrier via disruption of tight junction proteins including ZO-1 and claudin 1^25^,^35^. Bacterial entry through the paracellular route, in addition to direct cellular invasion^35^, is thought to be an important contributor to dissemination of infection^36^. In uninfected mouse ceca (VC mice or MET-1 mice), ZO-1 immunofluorescence was abundant in epithelial cells (Fig. 8a). In contrast, VC + *S*. Tm. mice had loss of ZO-1 membrane staining which was preserved in MET-1 + *S*. Tm. mice. Western blot analysis of the membrane-associated fraction of intestinal lysates demonstrated increased expression of ZO-1 in MET-1 + *S*. Tm. mice compared to VC + *S*. Tm. mice (Fig. 8b), indicating preserved expression of ZO-1 in MET-1 + *S*. Tm. mice.

Similarly, VC + *S*. Tm. mice showed reduced staining intensity, as well as disaggregation and clumping of epithelial claudin-1 immunofluorescence. This outcome was markedly attenuated in MET-1 + *S*. Tm. Mice (Fig. 8c,d). Additionally, Western blot analysis showed that MET-1 + *S*. Tm. mice had increased claudin-1 expression in the membrane fractions compared to VC + *S*. Tm. mice (Fig. 8d), showing that administration of MET-1 in *S. typhimurium-*infected mice preserved claudin-1 expression.

## Discussion

This study sought to address whether a defined gut microbial community that has been previously used to cure *C. difficile* infection^23^ may also act to protect against *S. typhimurium*, another gut pathogen and important cause of diarrheal illness in humans associated with antibiotic use and dysbiosis. We hypothesized that MET-1 would attenuate *S. typhimurium* infection through both antagonistic effects on the pathogen as well as effects on the host. However, we found that MET-1 had little effect on pathogen burden. Instead, MET-1 attenuated the systemic and localized effects of *Salmonella* infection and preserved intestinal tight junction protein expression.

MET-1 did not decrease *S. typhimurium* levels in the colon but systemic colonization was reduced in the infected animals treated with MET-1; levels of *S. typhimurium* in the small intestine were not measured, since MET-1 bacteria are derived from large intestine and are not expected to colonize the small intestine. However, the small intestine is a major point of *Salmonella* invasion and another study by Antunes *et al.* reported that fecal microbiota can inhibit the expression of invasion virulence factors by *S. typhimurium*^26^. In contrast to the latter report^26^, MET-1 did not have any effects on the ability of *S. typhimurium* to intracellularly invade intestinal epithelial cells when tested in an *in vitro* invasion assay. This may be due to the fact that the Lachnospiraceae species found by that study to be most inhibitory, such as *C. citroniae* and *C. aldenense*, are found within the *C. clostridioforme* subclade of the Lachnospiraceae family, and this group is not represented in MET-1. Collectively, our results indicate that MET-1 confers protection against *S. typhimurium* primarily through its effects on multiple host functions, rather than through direct effects on *Salmonella.*

MET-1 was found to have only modest inhibition of *Salmonella* in time-kill assays and no effect *in vivo.* The interplay between MET-1, *S. typhimurium*, and the host that occurs in the setting of the host inflammatory response to infection also likely contributes to this finding. Several studies have described the fitness advantage provided to facultative anaerobes in the environment of the inflamed intestine, replete with superoxide radicals, reactive oxygen species and reactive nitrogen species that may allow *S. typhimurium* to utilize alternate metabolic pathways and energy sources^37^,^38^. *S. typhimurium* has developed evolutionary strategies to utilize compounds reacting with organic sulfides and tertiary amines in the gut lumen as terminal electron acceptors for anaerobic respiration, thus allowing it to thrive in the gut under conditions of inflammation. Since MET-1 is composed primarily of anaerobic bacteria that cannot utilize these same metabolic pathways and carbon sources, we speculate that the inflamed milieu would provide favorable conditions for growth of *S. typhimurium* and may additionally contribute to the lack of MET-1 inhibition against *S. typhimurium* growth observed *in vivo*.

At the apical intestinal surface, TJ complexes between epithelial cells that involve cytoplasmic and membrane proteins such as claudin-1 and ZO-1 normally maintain the epithelial barrier^39^. By disrupting this, and altering the distribution of TJ proteins^40^ *S. typhimurium* can increase paracellular permeability and membrane “leakiness” to provide an additional route of access to the basolateral compartment^35^,^36^. Our data indicate that MET-1-mediated preservation of tight junction protein expression is a probable mechanism for the decreased systemic effects of *S. typhimurium*. While upregulation of mucins such as MUC2 by probiotic commensal bacteria has been reported^34^,^41^, we found no evidence to support a MET-1 effect on intestinal mucus or MUC2. We chose to focus on MUC2, since this is the main mucin produced by goblet cells^42^ however goblet cells produce other mucins that could potentially be induced by exposure to MET-1 and the possibility exists that other mucins or other secretory products from goblet cells, induced by MET-1, may exist that could contribute to host protection against *Salmonella*.

There was a significant reduction in the serum cytokines involved in the maturation and recruitment of neutrophils (GM-CSF, MCP-1 eotaxin and MIP-1β) in infected animals that received MET-1. It is tempting to speculate that a MET-1-mediated decrease in the neutrophil population in the gut of infected MET-1 mice may have also contributed to decreased barrier disruption, since neutrophil transmigration across the epithelial barrier causes a breach in barrier function through release of neutrophil proteases such as elastase, which transiently degrade epithelial tight junctions and increase “leakiness” across the paracellular space^43^,^44^.

In assessing the microbiome of MET-1 treated and non-treated groups we found that some MET-1 isolates were identical to murine microbiota sequences that pre-existed in the mice (such as the *Parabacteroides*), whereas others (*Acidaminococcus intestini, Roseburia faecalis/intestinalis, Bacteroides ovatus*, and *Bifidobacterium adolescentis/longum*) were unique to MET-1 and separable from what was originally in the mice at baseline, prior to receiving MET-1. However, limitations of the 16S rRNA gene profiling technique (which have been recognized in our previous human studies^23^) mean that it is impossible to be certain that changes in the murine gut microbiota brought about by MET-1 treatment were the result of colonization by MET-1 components; to effectively do this would require isolation of microbes from the murine gut, genome sequencing of these isolates, and alignment of obtained sequences with MET-1 component genomes, which was beyond the scope of this work. We are currently working to identify unique sequence signatures in MET-1 genomes that can be used to differentiate MET-1 strains from others and which can be used in our future efforts to understand the nature of MET-1 component colonization in different hosts.

As a complex ecosystem, MET-1 has the potential to accomplish multiple core functions of the microbiota and to act through multiple mechanisms to protect the host. Here, a single dose of MET-1 resulted in improved outcomes - a potential advantage of using a defined, gut-derived microbial ecosystem acting through multiple mechanisms, over standard single-strain probiotics^45^,^46^. This study extends previous observations using defined microbial mixtures to treat recurrent *C. difficile* infection^23^,^47^,^48^. We demonstrate here that MET-1 confers protective properties in *S. typhimurium* infection both through attenuated inflammation and preservation of tight junction proteins, providing evidence that this defined microbial community may be useful for the treatment of other enteric infections in addition to *C. difficile* infection. Future studies of the complex interplay between host, microbiota and pathogen will be instrumental in leading to both a better understanding of gastrointestinal infectious disease and novel strategies of microbiota-based therapies.

## Materials and Methods

### Ethics statement

This study was carried out in accordance with the guidelines of the Canadian Council of Animal Care and was approved by Queen's University Animal Care Committee.

### Bacterial culture

*Salmonella enterica* serovar Typhimurium SL1344 (Caliper Life Sciences, MA, USA), a streptomycin-resistant strain, was grown in Luria-Bertani (LB) broth (Bioshop Canada Inc., ON, Canada) supplemented with 100 μg/mL streptomycin at 37 ^o^C for 18  hours as previously described^49^.

The derivation of MET-1 (Table 1), commensal colonic bacteria from a healthy human volunteer, is described elsewhere^23^. In brief, the 33 MET-1 isolates were cultured on fastidious anaerobe agar (FAA) (Lab M Ltd., Lancashire, UK) with or without 5% defibrinated sheep blood (Hemostat Laboratories, CA, USA) under anaerobic conditions. Plates were incubated at 37 °C for 2–3 days under strict anaerobic conditions in a Bugbox (Ruskinn, Maine, USA). Biomass was scraped directly into pre-reduced, filter-sterilized 0.9% saline using microbiological loops to achieve 3.5 × 10^9^ CFU/mL^23^, previously determined to approximate the relative abundance of similar species in the stool samples of healthy North Americans (http://www.hmpdacc.org/).

### *Salmonella enterica* serovar Typhimurium colitis model

The *S. typhimurium* model of colitis^21^ was initiated in 6–7 week old female C57BL/6 mice (Charles River, MA, USA). Mice were fasted for four hours prior to oral gavage with 20 mg of streptomycin (SteriMax, ON, Canada), after which mice had access to food and water ad libitum. Eighteen hours later mice were gavaged with either 100 μL of MET-1 (corresponding to 3.5 × 10^8^ CFU) or vehicle control (0.9% saline). Four hours later mice were gavaged with 10^8^ CFU of *S. typhimurium* or phosphate buffered saline (PBS). Weights were monitored daily and mice from each treatment group were euthanized 48 hours post-infection. Tissues and blood were collected for analysis as described below.

### Bacterial enumeration in tissues and stool

Forty-eight hours post-infection spleens and colons were harvested for enumeration of *S. typhimurium*. Tissues were weighed, homogenized in 1 mL of sterile PBS, and serial dilutions of homogenates plated on MacConkey agar containing 100 μg/mL streptomycin, incubated at 37 °C for 24 hours. Colonies were confirmed as *S. typhimurium* by PCR and an amplified band of the correct size on a 1% agarose gel was confirmed to be from *S. typhimurium*^50^. PCR primer sequences used^50^ were: forward primer 5′-AACAACGGCTCCGGTAATGA-3′ and reverse primer 5′-TGACAAACTCTTGATTCTGA-3′, targeting a putative cytoplasmic protein (310 bp product, STM4497) and forward primer 5′-TTTGGCGGCGCAGGCGATTC and reverse primer 5′ GCCTCCGCCTCATCAATCCG (423 bp product, STM3098), targeting a putative transcriptional regulator, both of which have been used to detect *Salmonella* species^51^. Cycling conditions: 95 °C for 3 min, (95 °C for 30 s, 58 °C for 30 s, 72 °C for 90 s) × 30; 72 °C for 5 min.

### Multiplex bead assay to measure serum cytokine levels

Blood was collected via cardiac puncture 48 hours post-infection and a mouse cytokine magnetic bead kit (Bio-Plex Pro Mouse Cytokine multiplex, Bio-Rad, CA, USA) was used to measure serum IL-1α, IL-1β, IL-2, IL-3, IL-4, IL-5, IL-6, IL-9, IL-10, IL-12 (p40), IL-12 (p70), IL-13, IL-17A, eotaxin, G-CSF, GM-CSF, INF-γ, KC, MCP-1, MIP-1α, MIP-1β, and TNF-α, as per the manufacturer’s instructions.

### Immunohistochemistry

Immunofluorescence staining was carried out on formalin-fixed cecal tissues with the exception of MUC2, where tissues were fixed in Carnoy’s fixative^52^. Paraffin embedded tissues were sectioned (4 μm), hydrated and subjected to antigen retrieval in citric acid buffer (10 mM citric buffer, 2 mM EDTA, 0.05% Tween 20, pH 6.0).

For myeloperoxidase (MPO), claudin and NF-κB P65 staining, tissues were blocked with PBS containing 0.3 M glycine and 0.1% Tween 20, 3% bovine serum albumin (Bioshop Canada Inc.) and 10% goat serum (Sigma Aldrich), for one hour at room temperature. For MUC2, tissues were blocked with PBS with 5% fetal bovine serum (FBS) for 1 hour at room temperature. ZO-1 tissues were blocked with PBS-T containing 2% bovine serum albumin and 1% goat serum for 1 hour at room temperature.

Sections were stained with rabbit anti-MPO (1:100, Abcam, MA, USA) or rabbit anti-claudin-1 (1:200, Abcam), or rabbit anti-NF-κB P65 (1:50, Santa Cruz) or rabbit anti-MUC2 (1:100 dilution, Santa Cruz) or 1:100 mouse anti-ZO-1 (Invitrogen), in blocking buffer over night at 4 °C. Sections were washed with PBS-T three times, then incubated with Alexa 488-conjugated goat anti-rabbit immunoglobulin G (Invitrogen, CA, USA) at a 1:500 dilution (MPO, claudin, NF-κB, and MUC2) or 1:100 (ZO-1) in blocking buffer. Tissues were incubated for one hour at room temperature for MPO, claudin, NF-κB, ZO-1 and two hours at room temperature for MUC2. Prolong antifade mounting media containing 4,6-diamidino-2-phenylindole (DAPI) was used to stain nuclei (Invitrogen).

For MPO, claudin and NF-κB, images were obtained using an Olympus BX51 microscope and Image-Pro Plus software (Media Cybernetics, Inc., MD, USA). MUC2 images were visualized using an AxioImager M.1 microscope and AxioVision software (Carl Zeiss, Oberkochen, Germany). ZO-1 images were obtained using a Zeiss LSM 710 Laser Scanning confocal microscope.

Images used for quantification were taken at 400× magnification from at least two non-adjacent sections of cecum per mouse, and 4 non-adjacent images were then used for each section to quantify. Images were taken from two independent experiments and each experiment had 3–4 mice per group. Total number of cells (determined by DAPI staining of nuclei) per view at 400× magnification was counted using ImagePro Plus software. An average of 700 cells (range: 540–1000 cells) was counted per view. The area of green fluorescence was measured using the ImagePro Plus software. The ratio of area of green versus total number of nuclei was used for the quantification^53^. Data were analyzed using a 1-way ANOVA with Bonferroni correction.

### Alcian blue staining

Sections of mouse ceca were rehydrated, immersed in 3% acetic acid for 2 minutes and then stained with 1% (w/v) Alcian blue solution (8GX, Acros Organics, NJ, USA) in 3% acetic acid (pH 2.5) for 30 minutes. Tissues were rinsed in distilled water and counterstained with 0.1% nuclear fast red solution (Acros Organics) for 1 minute. All images were visualized using an Olympus BX60 microscope and Infinity Capture software (Lumenera Corporation, ON, Canada). Goblet cells were enumerated from stained tissues by counting goblet cells in 10 random high-powered fields/sample (400x magnification) spanning muscularis mucosa to surface epithelium.

### Western blot analysis

Cecal tissues were sonicated in lysis buffer (10mM Tris, pH 7.6 containing 5 mM MgSO_4_, DNAse I, RNAse A, and protease inhibitor cocktail; Roche, ON, Canada). Samples were incubated on ice for 20 minutes and centrifuged at 2,900 × g for 20 minutes at 4 °C. For fractionated samples, supernatant containing both cytosolic and membrane fractions were centrifuged at 21,000 × g for 45 minutes at 4 °C. The resulting supernatants comprised the soluble (S) cytosolic fractions. Pellets were resuspended in RIPA buffer (25 mM Tris, pH 7.6, 150 mM NaCl, 1% NP-40, 1% sodium deoxycholate, 0.1% SDS, and protease inhibitor cocktail), left on ice for 20 minutes, and centrifuged at 15,000 × g for 20 minutes at 4 °C; the resulting supernatants comprised the insoluble (I) membrane fractions.

Samples of whole lysates, soluble fractions, or insoluble fractions were separated on a 12% SDS-PAGE gel, transferred overnight before blocking with 5% non-fat dry milk in TBST for 1 hour at room temperature. Membranes were then incubated with 1:1000 anti-ZO-1 (Life Technologies, ON, Canada), 1:1000 anti-claudin-1 (Life Technologies), or 1:10,000 anti-β-actin (Sigma Aldrich) overnight at 4 °C. After washing with TBS-T three times, horseradish peroxidase (HRP)-conjugated secondary antibodies were added (1:10,000 dilution, Cell Signaling, MA, USA) and membranes incubated for 1 hour at room temperature. After three more washes with TBS-T, PVDF membranes were exposed to chemiluminescence (ECL) reagents (Thermo Fisher Scientific, MA, USA) and exposed to autoradiographic film (VWR, ON, Canada) for band visualization. Protein levels were quantified by densitometry using ImageJ software.

### Salmonella enterica serovar Typhimurium time-kill assay

MET-1 (initially 3.5 × 10^9 ^CFU/mL) was co-cultured with *S. typhimurium* (using different dilutions of MET-1, see below) and *S. typhimurium* viable counts monitored over time. Each well contained 10^7^ CFU *S. typhimurium* in Wilkins Chalgren broth (Bioshop Canada Inc.) The overall bacterial CFU of MET-1: *S*. Tm. were as follows: MET-1 1:2 dilution = 3.5 × 10^9^ CFU MET-1: *S. typhimurium* (350:1); 1:100 dilution = 7.0 × 10^7^ CFU MET-1: *S. typhimurium* (7:1); 1:300 dilution = 2.3 × 10^7 ^CFU MET-1: *S. typhimurium* (2.3:1). Mixtures were incubated at 37 °C under anaerobic conditions (90% N_2_, 5% CO_2_, and 5% H_2_) for 24 hours, and 100 μL aliquots withdrawn at 0, 1, 2, 3, 4, 5, 6, 7, 8, 12, and 24 hours, serial diluted, and plated on MacConkey agar plates containing 100 μg/mL streptomycin. Plates were incubated at 37 °C for 24 hours and colonies were enumerated.

### Salmonella enterica serovar Typhimurium invasion assay

The effect of MET-1 pretreatment on the susceptibility of Caco-2 cells (ATCC HTB-37) to *S. typhimurium* infection was assessed using an invasion assay based on a previous study^54^. Briefly, Caco-2 cells were seeded in 12-well plates and maintained in Dulbecco’s minimal essential medium (DMEM) supplemented with 10% fetal bovine serum (FBS) (37 °C in a 5% CO_2_, 95% air atmosphere). Wells containing 70–90% confluent monolayers^54^ were washed in DMEM and MET-1 or saline was added to each well (10% concentration). Cells were incubated at 37 °C for 1 hr, media were removed, cells were washed with PBS, and fresh media were added. Washed live or heat-killed (boiled for 30 min) *S. typhimurium* (multiplicity of infection of 100:1) or saline (equivalent volume) were added to each well. Cells were incubated at 37 °C for 1 hr and washed with PBS. Non-internalized *S. typhimurium* cells were killed with gentamicin (100 μg/mL) for 1 hr at 37 °C. Media were removed, cells were washed with PBS, and cells were treated with 0.1% Triton X-100 and incubated for 10 minutes. CFU/mL of *S. typhimurium* were calculated by serial dilutions plated onto MacConkey agar (Difco Laboratories, Becton, Dickinson and Company, MD, USA) supplemented with streptomycin (100 μg/mL).

### Quantitative real-time PCR

An approximately 5 mm section of cecum tissue was immediately stored in RNAlater (Sigma Aldrich). Briefly, tissue was homogenized and RNA was extracted using an RNeasy Mini kit (Qiagen, ON, Canada) as per manufacturer’s instructions. RNA quality was assessed by absorbance at 260 nm (on a spectrophotometer) and by running on the Agilent 2100 Bioanalyzer (Agilent Technologies, ON, Canada). RNA was treated with RNAse free DNAse I (Invitrogen) to eliminate genomic DNA and quantitative RT-PCR (qRT-PCR) was performed in triplicates using the StepOnePlus^TM^ Real-Time PCR System (Life Technologies). qRT-PCR was run using q-Script^TM^ One-Step SYBR Green qRT-PCR kit (Quanta Biosciences, MD, USA) in a 10 μL reaction volume containing equal amounts of RNA (62 ng), 5 μL One-Step SYBR Green Master Mix, 0.2 μL qScript One-Step Reverse Transcriptase, and gene specific forward (TNF-α, 200 nM; GAPDH 50 nM) and reverse (TNF-α, 50 nM; GAPDH, 150 nM) primers. Cycling conditions were as follows: 50 °C for 5 min, 95 °C for 2 min, (95 °C for 3 s, 60 °C for 30 s) × 40 cycles. The TNF-α and GAPDH primer sequences were as follows: TNF-α, forward 5′-CCACCACGCTCTTCTGTCTA-3′, reverse 5′-AGGGTCTGGGCCATAGAACT-3′; GAPDH, forward 5′-CGTCCCGTAGACAAAATGGT-3, reverse 5′-TTGATGGCAACAATCTCCAC-3′. The relative expression of TNF-α normalized to the expression of GAPDH (endogenous control) was calculated by the comparative C_T_ (ΔΔC_T_) method^55^ using the vehicle control group as a reference group and one of the vehicle control group mice as a reference sample.

### Statistical analysis

Results are expressed as the mean value with standard error of the mean (SEM). Statistical analyses were performed with GraphPad Prism version 5.0 (GraphPad Software, CA, USA). A two-tailed unpaired t-test, 2-way ANOVA with a Bonferroni correction, or 1-way ANOVA with Newman-Keuls or a Bonferroni correction was used where indicated. Statistical significance was set at a P value of <0.05.

## Additional Information

**How to cite this article**: Martz, S.-L. E. *et al.* Administration of defined microbiota is protective in a murine *Salmonella* infection model. *Sci. Rep.* **5**, 16094; doi: 10.1038/srep16094 (2015).

## Supplementary Material

Supplementary info - Fig.S1

## Figures and Tables

**Table 1:** List of cultured isolates from the healthy donor comprising the MET-1 synthetic community. Based on their DNA sequence identification, the MET-1 strains are represented according to their phylogenetic nomenclature/biological classification (http://www.arb-silva.de/projects/living-tree/)^56^.

	Higher taxonomic group	Closest species match^a^
	*Actinobacteria*	*Bifidobacterium adolescentis* (two different strains)
		*Bifidobacterium longum* (two different strains)
		*Collinsella aerofaciens*
	*Bacteroidetes*	*Bacteroides ovatus*
		*Parabacteroides distasonis*
*Firmicutes*	*Bacilli*	*Lactobacillus casei/paracasei*
		*Lactobacillus casei*
		*Streptococcus mitis*
	*Clostridium* cluster IV	*Eubacterium desmolans*
		*Faecalibacterium prausnitzii*
	*Clostridium* cluster VIII	*Clostridium cocleatum*
	*Clostridium* cluster IX	*Acidaminococcus intestini*
		*Blautia sp.*
		*Dorea longicatena* (two different strains)
		*Eubacterium eligens*
	*Clostridium* cluster XIVa	*Eubacterium rectale* (four different strains)
		*Eubacterium ventriosum*
		*Lachnospira pectinoshiza*
		*Roseburia faecalis*
		*Roseburia intestinalis*
		*Ruminococcus torques* (two different strains)
		*Ruminococcus* spp. (two different strains)
	*Clostridium* cluster XV	*Eubacterium limosum*
	*Proteobacteria*	*Escherichia coli*
		*Raoultella* sp.

The table has the 4 major gut phyla (Actinobacteria, Bacteroidetes, Firmicutes, and Proteobacteria), with the Firmicutes further divided into classes, for clarity.

^a^Closest species match inferred by alignment of 16S rRNA sequence to GreenGenes database.
