# Supplementary material for: Administration of defined microbiota is protective in a murine Salmonella infection model
Source: Sci Rep. 2015 Nov 4;5:16094. doi: 10.1038/srep16094 (PMC4632038; doi:10.1038/srep16094)
Supplement: Supplementary info - Fig.S1 [file srep16094-s1.pdf]

Supplementary Data for

“Administration of defined microbiota is protective in a murine *Salmonella* infection model”

Sarah-Lynn E. Martz[1], Julie A. K. McDonald[1], Jun Sun[2], Yong-guo Zhang[2], Gregory B Gloor[3], Curtis Noordhof[1], Shu-Mei He[1], Teklu Gerbaba[1], Michael Blennerhassett[1], David J. Hurlbut[4], Emma Allen-Vercoe[5], Erika C. Claud[6], and Elaine O. Petrof[1#]

[1]Dept. Medicine, Gastrointestinal Diseases Research Unit, Queen’s University, Kingston, ON, K7L 2V7, Canada

[2]Dept. Biochemistry, Rush University Medical Center, Chicago, IL, 60612, USA

[3]Dept. Biochemistry, University of Western Ontario, London, Ontario, N6A 5C1, Canada

[4]Dept. Pathology & Molecular Medicine, Queen’s University, Kingston, ON, K7L 2V7, Canada

[5]Dept. Molecular & Cellular Biology, University of Guelph, Guelph, ON, N1G 2W1, Canada

[6]Depts. Pediatrics and Medicine, University of Chicago, Chicago, IL, 60637, USA

Supplementary File – Figure S1

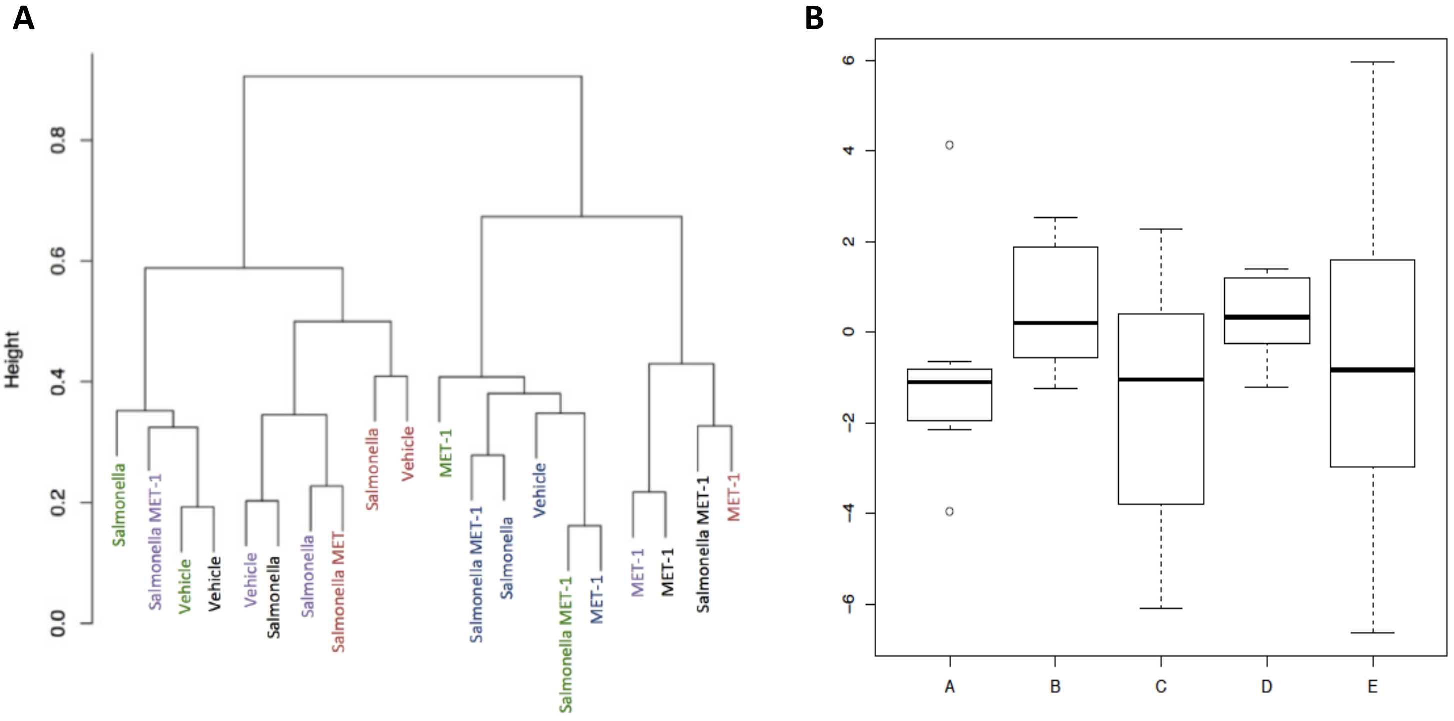

Fig S1. (a) Dendrogram of the similarity between the different groups based on weighted unifrac distance. The microbial composition of fecal pellets of mice from 5 independent experiments were collected from the cages of the different treatment groups (run 1 = blue, run 2 = red, run 3 = green, run 4 = purple, run 5 = black). The Y- axis indicates weighted unifrac distances between samples calculated from relative abundance of operational taxonomic units (OTUs). This indicates similarity between samples at the operational taxonomic unit level (OTU) weighted by the proportional abundance of each OTU. VC = uninfected mice pretreated with vehicle control ; MET-1 = uninfected mice pretreated with MET-1; VC + S. Tm. = S. typhimurium-infected mice pretreated with vehicle control; MET-1 + S. Tm. = S. typhimurium-infected mice pretreated with MET-1. (b) Box plot of differences between MET-1 OTU abundances in five replicates of mice treated with Salmonella + MET-1, vs Salmonella + vehicle. The representative OTU sequences were compared by BLAST to the 16S rRNA gene sequences of the MET-1 isolates, and seven OTUs were found with sequences that that were 100% identical to the MET-1 isolates. These OTUs represent the following Phyla; Genera: Bacteroidetes; Parabacteroides, Bacteroidetes; Bacteroides, Firmicutes; Pseudobutyrivibrio, Actinobacteria; Bifidobacterium, Firmicutes; Acidaminococcus, Firmicutes; Eubacterium, Firmicutes; Incertae_Sedis. Abundances were log ratio transformed, and the differences between the two groups for the seven genera determined. The genera were also observed in vehicle control at approximately the same abundance as in the MET-1 treated animals, suggesting that at the resolution available in 16S rRNA gene sequencing there was little difference in abundance. Replicates are indicated as A-E. The y-axis is on a log(2) scale.
